# Toward building deliberative digital media: From subversion to consensus

**DOI:** 10.1093/pnasnexus/pgae407

**Published:** 2024-10-15

**Authors:** Alex Pentland, Lily Tsai

**Affiliations:** Stanford Digital Economy Lab, Stanford University, 353 Jane Stanford Way, Mail Code: 4115, Stanford, CA 94301, USA; GovLab, Massachusetts Institute of Technology, 30 Wadsworth Street, Cambridge, MA 02142, USA

**Keywords:** digital media, polarization, deliberative democracy, misinformation, consensus

## Abstract

Evidence-based and human-centric design of digital media platforms could reduce many of the problems of misinformation, polarization, and misaligned incentives that plague both society and individual organizations. With these sorts of design changes, it may become possible to build deliberative digital media that are useful both for discussions of contentious issues and for achieving successful collective action. In this Perspective paper, we discuss several issues in which current-day social science indicates the origin of these problems and suggests methods for improvement. Finally, we analyze a popular deliberative democracy platform to illustrate how social science might enable design of next-generation digital media suitable for democratic deliberation, and in which generative artificial intelligence might be useful.

## Introduction

Political polarization and mistrust of government has increased all over the world, undermining democratic governance and contributing to the rise of authoritarian leaders. There is strong evidence that digital media, and particularly social media, are contributing to this problem. While social media have given exposure to many previously unheard voices, research has shown that the current social media platforms also contribute to several problems. Some of the areas in which there is already substantial understanding of these problems include the following:

- Misinformation, and associated behavioral cascades (“herding”)
- Misperception of intent, and associated polarization, especially affective polarization
- Misaligned incentives that hinder inclusive democratic deliberation

And, of course, research on social media is still evolving. In this paper, we examine these three areas in turn and explore practical methods of improving the design of digital media in general and the design of digital deliberation platforms in particular. We then illustrate how these methods might work, or fail to work, by analyzing a popular deliberative democracy platform. Finally, we observe that generative artificial intelligence (GenAI) could dramatically worsen some of these problems, especially for the critical function of democratic governance, and that we need to adopt certain “north star” principles to guide incorporation of GenAI into our society's governance mechanisms.

### Social science insights for digital platform design

In this section, we review social science insights that potentially provide guidance for the design of deliberative digital media. In the following section, we illustrate the use of these insights in critiquing a popular platform for democratic deliberation.

#### Misinformation

In the last decade, society at large has become very concerned about the rapid spread of misinformation. It is unclear how the prevalence and effects of misinformation today compares with the situation in previous generations. For instance, just before the emergence of social media, the fraction of US citizens that believed that the sun circles around the earth (1) was larger than the fraction that still does not believe in global warming (2).

However, the misinformation problem has changed greatly in one very significant way: false information now spreads much more quickly (3). This rapid dissemination of misinformation likely serves to undermine the authority of statements by government experts and scientists to a greater degree than previously because the misinformation is often encountered while citizens are still considering the expert statements, rather than being encountered afterward. During the 2020 COVID pandemic, this seems to have made successful collective action more difficult and made efforts to suppress opposing views more tempting.

The acceleration of misinformation spreading is likely due to the design of digital media platforms and business models that reward impulsive sharing. Many researchers [e.g. (4)], have shown that interfaces that prompt people to reflect about accuracy before sharing can dramatically reduce the spread of misinformation. A recent MIT PhD thesis showed that this is likely a System 1 vs System 2 phenomenon (5). Immediate reactions to new stimuli are typically mediated by System 1, such as alerting behaviors when signs of danger are detected, whereas more reflective judgments are mediated by the slower but more sophisticated cognitive mechanisms of System 2 (6).

The practical implication is that platforms intended for informing people and supporting deliberation should not offer instant reposting of information, but instead should adopt an interface that inserts an extra step or provides a prompt that promotes reflection before the user chooses to share information. This “pause and reflect” intervention has been shown to significantly reduce misinformation spread (4) but typically will impact the profitability of platforms based on maximizing engagement. This may mean that most current digital media platforms are unsuitable for deliberation and discussion of controversial topics.

#### Misperception of intent

Trust is central to most social functions and is especially important for democratic deliberation and collective action. For instance, citizens in a democracy must be willing both to trust that compromises are fair and that others are not cheating [e.g. (7)]. Undermining trust is a strong temptations for authoritarians, because by spreading fears about potential opponents’ fair dealing, they can seize greater power and gain acceptance of undemocratic and even unethical behaviors (8, 9).

In our recent paper exploring this problem (10), we called this the “subversion dilemma” because it has parallels to the “security dilemma” that is central to the realist view of international relations [e.g. (11)]. In the realist view, fear incentivizes one country to arm itself against potential attackers. This military buildup can be seen by other countries as a threat, and they may in turn increase their own armaments. The first country then reacts to their buildup by increasing their military funding even more, and this cycle may continue until one state decides to preemptively attack because of fear of annihilation. The result is that warfare breaks out even though everyone prefers to avoid the costs of war.

Similarly, partisan groups can fall into a cycle of escalating fear and increasing perceptions of threat. Authoritarians’ ability to use fear of others is a key aspect of mutually reinforcing polarization, including increased partisan identity strength, heightened dislike, and increased dehumanization [e.g. (12)].

In a demographically balanced, large-scale examination of this cycle of increasing fear and loathing in the United States, Braley et al. (10) found that when people knew more about the intentions of the other side, they had more trust that everyone was abiding by democratic norms, and they more frequently voted for democracy-promoting candidates. In international relations research into the security dilemma, it has been found that in many cases third-party observation of behaviors that signal partisan intentions, along with signals of good-faith intentions, are possible ways to resolve the dilemma (11). Research in political science has also shown that costly signals of good intentions build trust and cooperation (13).

This idea that mutual knowledge among partisans, especially knowledge about intentions, was further tested in a recent megastudy (14) that involved 32,059 subjects to test 25 interventions designed by leading academics and practitioners. Interventions that corrected misperceptions about the intentions of opposing partisans were surprisingly effective in reducing support for undemocratic practices. Because the online testing environment of this megastudy resembled typical online environments, it is reasonable to expect similar effects for many online applications. For example, this study found that a short, gamified question-and-answer session about the intentions of opposing partisans led to a significant reduction in misperceptions and increased support for democratic policies (10, 14).

#### Misaligned incentives

Some of the most persistent problems with using digital media for deliberation and collective action are misaligned incentives. As mentioned previously, most current digital media platforms make money off of user engagement, and so they encourage attention-grabbing posts that will likely be reposted, which may make them inappropriate for deliberation. Similarly, political figures sometimes claim that opponents have an ill intent in order to strengthen their political base. Extreme claims are often reposted, again raising the profits of most digital platforms.

In addition to misalignment of the monetary incentives of digital media with the goal of productive discussions, there are also problems of misalignment with individuals’ incentives. Three important examples are (1) people who interject comments that distract from prosocial, civil, and useful community conversations; (2) the dominance of discussions by a few “influencers” or similar disproportionately dominant voices; and (3) lack of participation by less wealthy and minority communities. These problems are, of course, interrelated and mutually reinforcing.

The first of these problems, disruption, is widely dealt with by moderation. Platforms such as Reddit, in which discussion forums often have strict guidelines, have human moderators that intervene to help reformulate comments so that they follow guidelines or, failing that, outright reject inappropriate comments. Similarly, platforms intended for digital deliberation often include strict moderation of comments to ensure that they are civil and helpful.

Unfortunately, human moderation is either expensive or requires unusually generous efforts by volunteers. Computers are commonly used for simple sorts of moderation, for instance, spotting of banned words, but until recently more sophisticated moderation was beyond their capabilities. Today, progress in large language models such as ChatGPT permit much more sophisticated and effective moderation without introducing biases, as shown in Argyle et al. (15).

The second problem is that of inequality of voice. Today's media companies and digital platforms like to represent themselves as platforms for people to express themselves and engage in the sort of civic discourse needed to improve quality of life and guide collective action. In reality, however, most people have very few followers and post only personal content for friends and family. Relatively few people have more than a few thousand followers, and a very small number have many millions.

This suggests that instead of being forums for sharing ideas and discussion, digital media are more like bazaars where sellers loudly hawk their goods and ideas. Moreover, those with the loudest voices drown out everyone else. In current social media platforms, the loudest voices also get more followers, resulting in a “rich get richer” feedback loop that means that the attention of people using the platform is dominated by just a few of the loudest voices. Importantly, most misinformation impressions come from a very small group of accounts with large numbers of followers.

In a recent paper in the *Proceedings of the National Academy of Sciences of the United States of America* (16), we mathematically demonstrated that platforms like today's social media will inevitably become dominated by a very few accounts with extremely large numbers of followers. The mathematical consequence of domination by the few is that conversations on these platforms are neither inclusive nor rational (17).

How can we avoid this problem? In our paper (16), we were able to show mathematically that the only stable solution of the problem is to reduce the “rich get richer” feedback so that that there is less inequality in participants’ ability to communicate their message to others. One common method of reducing inequality is to dampen the “rich get richer” feedback loop is through progressive taxation. For instance, imagine that we taxed influential media accounts in proportion to the number of “followers” they have (perhaps letting the first 10,000 followers be free). This could help level the playing ground for individuals and local organizations so that digital media could play a much more positive role in society.

People worry that taxation of social media accounts according to the number of users poses a freedom of speech problem. We believe that it does not pose an insuperable problem, because by any common-sense definition, these “influencer” accounts are a type of business, whether they are formally incorporated, or for profit, or not. People confuse “free delivery” with “freedom of speech,” despite the fact that TV stations have always had to buy licenses, cell phone companies have to buy wireless spectrum, and magazines pay for postal delivery. Businesses have always paid to use the public commons, so why not digital media? A legal analysis of this potential solution can be found in Mahari et al (18).

The third of these problems, which is lack of participation by marginalized communities, may be largely a problem of misaligned incentives. Why should members of marginalized communities incur cost in time, attention, and data fees to contribute to discussions in which they have little influence? Why should members of wealthy or majority communities spend their time and attention engaging with the concerns of marginalized communities? There is little incentive for real engagement beyond general altruism and idealism.

For some critical communication functions, such as democratic voting, nations such as Australia and Brazil address this incentive problem by mandating universal participation with fines for noncompliance. These mandates are moderately successful for promoting the physical action of voting but do not address the sort of time-consuming, thoughtful discussion required for deliberative democracy. Consequently, even with universal voting marginalized communities still have little influence on outcomes.

An intriguing observation is that the part of the population that does not participate, whether from lack of resources or because they feel that they will not be listened to, is similar to the population that participates in lotteries and activities like sports betting. Use of lottery-style incentives to encourage civic participation has shown promise in both breadth of participation and depth of deliberation (19). This study found that extrinsic rewards boosted voting significantly and that the effects of a lottery appear to be especially strong among those of lower socioeconomic status. Importantly, the effect is probably not primarily driven by economic considerations, as the average return is quite small, but rather by the social nature of a multiperson game. This will be discussed further in the following sections.

This research shows improvement in voting participation among a population that normally has low voting participation, but more study is required to prove the causality and effectiveness of lottery incentives in a general population. A related, better studied intervention is the British lottery bond system, in which people who save money by purchasing bonds are automatically entered into a national lottery. A large percentage of all British adults participate in this program.

In both the La Raja and Schaffner study (19) and the British lottery bond system, the lottery creates only an incentive to participate but does not create an incentive for picking a policy (from a set of suggested policies) that may eventually become law. In the context of a democratic deliberation platform like Polis, it is easy to tie the number of lottery tickets you receive to the eventual popularity of the policy you pick.

The connection between chances of winning the lottery and the popularity of the policies that you support constitutes a prediction market, in which the most likely outcomes are predicted by market dynamics. As pointed out by Nobel Prize laurate Kenneth Arrow, prediction markets have long shown promise in predicting political outcomes and can be a key part of building democratic consensus (20).

Our experiments have shown that incentives cast as social games are dramatically more effective than equivalently sized direct economic incentives (21). In China, online versions of similar social incentives, with hundreds of millions of participants, have been shown to reinforce social ties and encourage greater participation (22).

### Using social science insights to design a platform for democratic deliberation

In the first section of this Perspective paper, we examined insights from social science research that suggest ways to improve digital media platforms generally, with the goal of designing better platforms for deliberation. In this section, we critique a popular democratic deliberation platform using these social science insights and suggest how GenAI might best be used to improve this sort of digital deliberation.

#### Toward digital democracy: an illustration

The rise of social media platforms intended for casual conversation and entertainment has been paralleled by the development of online technologies to share opinions on policy questions and seek consensus on recommendations. These “deliberative platforms” use interfaces and technologies that are different from conventional methods of public deliberation and promote themselves as being able to achieve deliberative goals faster, more inclusively, and at a larger scale, while at the same time minimizing human bias and costs.

One online platform that attempts to harness digital media for deliberative democracy is the Polis system, which is frequently cited as being among the most effective direct democracy social media tool and has been successfully used by governments on 3 continents (23). It allows governments to pose policy questions and use simple statistical methods to provide graphical feedback on citizen beliefs and desires in order to promote consensus. Press coverage of Polis claims that it is effective at achieving consensus among citizens even on contentious issues within 2 or 3 weeks. Consequently, it is interesting to analyze how Polis differs from typical social media platforms and consider if some of these differences could be used to reform social media.

The way Polis operates is that a topic is put up for debate, and then citizens who create an (verified, but anonymous) account can anonymously post comments and upvote or downvote other people's comments. Unusually, users cannot reply to comments directly, making it difficult to engage in flame wars and trolling. The comments and upvote/downvote mechanism create a citation network, similar to citation networks that are the main archival mechanism used to organize knowledge in scientific papers, patent applications, and legal decisions. In Polis, the citation network records the comments of citizens and their approval/disapproval of other people's comments, and it is this record of how the collective spectrum of opinions are evolving that drives interaction with citizens and not the actual content of the comments.

The pattern of upvotes and downvotes is used by Polis to generate a visualization of all the comments, clustering together people who have similar up and down votes. Although there may be a huge number of comments, similar comments cluster together, showing the populations’ divides and areas of consensus. The Polis framing encourages people to make comments that attract many upvotes, and this may be the explanation for why people try to draft comments that will win votes from both sides of a divide, gradually eliminating the gaps.

The Polis citation mechanism provides two important constraints that are likely critical to the success of the system. One constraint is that each individual can make only a very few comments per day. Moreover, while comments are submitted anonymously, they must be made by a human whose identity and right to participate has been verified. This means that influencers, political campaigns, and companies cannot have the disproportionate influence that they have in regular social media. This serves to prevent them from dominating the conversation (16). The second constraint is that people are required to upvote and downvote other people's comments before submitting their own comment. The process of surveying people's comments for the purpose of upvoting and downvoting means that people will learn about others’ opinions, and this tends to promote “wisdom of the crowd” effects and promotes better decision making (24).

The Polis visualization of up- and downvoting of the comments seems to be very helpful in promoting convergence of opinion, and is much like the visualizations that have proven very effective in domains such as finance (25). Audrey Tang, the Taiwanese Minister of IT, said (23), “If you show people the face of the crowd, and if you take away the reply button, then people stop wasting time on the divisive statements.”

Computational social science research within the Pentland lab shows that there is reason to believe that the Polis-style “face of the crowd” approach will have a very significant impact on decreasing polarization. In addition to the “what do others think” feedback system tested in the subversion dilemma paper (10), other Pentland lab experiments have shown that providing users with a visualization of the range of other people's opinions and actions improves financial decisions (17, 24, 25).

#### Ethical considerations for use of GenAI

New technologies such as GenAI can potentially change the fundamental nature of how online deliberation on policy issues occurs, and many people are experimenting with ways of integrating GenAI into digital media platforms [e.g. (15)]. As discussed in the previous section, the interface of these early deliberation platforms includes open commenting, up- and downvoting, and simple polling, and they often organize deliberations in nontraditional ways including citizen assemblies and participatory budgeting. Some platforms, such as Polis, are beginning to use machine learning techniques to generate visualizations for users, to show areas of agreement, disagreement, and groupings in order to promote consensus.

These innovations raise important questions about how to uphold individual rights while using these new technologies, and especially in the case of GenAI technologies. Given our society's commitment to democratic ideals, the most urgent of these questions are how best to use GenAI technologies.

In our recent white paper on the subject (26), we offered a framework for thinking about how to design democratic deliberation platforms that use GenAI. Our white paper assessed the opportunities and risks of GenAI in terms of this framework and identified the most promising directions for GenAI to support online discussion and deliberation. The 4 principles of this framework suggest that (1) GenAI should assist citizens without reducing their agency; (2) GenAI should treat citizens as political equals and enable citizens to treat each other with mutual respect; (3) GenAI should both protect citizens from harm by biased algorithms or bad actors while also engendering the trust essential for participation in democratic deliberation; and (4) AIs should not perform our responsibilities as citizens on our behalf, or serve as our representatives in policy deliberation or policymaking processes.

Specifically, we see the potential to improve online deliberative democracy platforms using GenAI to support the strength of engaged deliberation and the ability to scale these platforms while upholding democratic ideals such as preserving human agency, mutual respect, equality and inclusiveness, and augmenting active citizenship.

#### GenAI approaches to improve the polis

There are several ways to apply GenAI to existing deliberative platforms that are in accordance with our AI governance framework. Simple AI tools have already been deployed on some social network platforms to remind users about community standards. Users seem to readily accept these reminders, and many deployments of Polis have either human or AI comment moderation. Perhaps most promising is the work of Argyle et al. (15), which used GenAI for moderation, which has been shown to be effective at reminding the user to be civil and prosocial when they are contributing comments, and also has been shown to not bias or influence user opinions.

Going further, researchers at DeepMind have fine-tuned a GenAI model to generate statements that maximize the expected agreement among a group of people with diverse opinions. They found that more than 65% of the time study participants preferred the GenAI consensus statements to those written by humans (27).

While simply presenting the AI generated consensus statement to participants may be “leading the witness” and thus reduce human agency, with the right interface and interaction it could be even more effective than the Argyle et al. (15) system. The performance of this consensus system also suggests the potential for GenAI to help diverse groups of people find agreement. For instance, a small variation on this DeepMind GenAI tool could encourage users to make more constructive and prosocial contributions to the discussion by providing estimates to participants on how many more people might agree with them if they amended their statement in one or two places.

Another promising area for improvement is better visualization of community opinion. Visualization is a key part of Polis, and a variety of visualization tools are widely used in both traditional and digital media to give users a better sense of context and to inform them about what other people think. GenAI tools are just beginning to be used to create more effective visualizations, and we think that this is an area of great promise for deliberative democracy platforms.

For instance, in our recent paper using AI-embedding techniques to analyze the evolution of science, patents, and law citation networks, we demonstrated the ability to visualize the evolution of the network to better predict community convergence (28). We expect that the same sort of visualization will be effective in helping citizens understand the evolution of opinion in a Polis-like platform. Such a visualization method could provide citizens the ability to think more clearly about the dynamics of political deliberation (e.g. which way the discussion is trending) and then formulate responses that are more effective in achieving their aims.

In summary, social science research has shown important directions for improved design for both general digital media platforms and deliberative democracy platforms. Experimental evaluation of both the idea of helping people craft more effective comments by using GenAI, and the idea of creating better visualizations to give users more context seems particularly promising. We expect that social science informed research will continue to provide insight into how to use AI to build healthier and more effective digital communities and help us protect against the most dangerous aspects of AI.

## Data statement

There are no data underlying this work.
